# Sex differences in the association between sarcopenia index and sarcopenia: a cross-sectional study from a Chinese community-based population

**DOI:** 10.1007/s41999-024-01111-w

**Published:** 2024-12-02

**Authors:** Hong Yang, Yunda Huang, Guihua Jiang, Zhiping Duan, Runfen Du, Yinan Hao, Wei Huang, Xiaoling Liu

**Affiliations:** 1 Department of Geriatrics, The Third People’s Hospital of Yunnan Province, 292 Beijing Road, Kunming, 650011 Yunnan China; 2 Radiotherapy Department, Yunnan Cancer Hospital, No. 519 Kunzhou Road, Kunming, 650118 Yunnan China

**Keywords:** Sarcopenia index, Creatinine, Cystatin C, Sarcopenia, CHARLS

## Abstract

**Purpose:**

The sarcopenia index (SI) is a convenient method to screen for sarcopenia, but few studies have analysed whether there are sex differences. The aim of this study was to analyse sex differences in the relationship between SI and sarcopenia in a Chinese community-based population.

**Methods:**

This cross-sectional study included participants from 2011 China Health and Retirement Longitudinal Study. The SI was defined as 100 × creatinine / cystatin C. Diagnosis of sarcopenia based on the Asian Working Group for Sarcopenia 2019 consensus. Logistic regression model, linear regression model, and natural spline model were used to analyze the association between SI and sarcopenia.

**Results:**

A total of 7,118 participants with a mean age of 60.6 ± 10.1 were included, 53.4% females. In males, the prevalence of sarcopenia decreased by 25% for every 10 increase in SI, and skeletal muscle mass index (SMI) increased by 0.04, odds ratio (OR), β, and 95% confidence interval (CI) were 0.75 (0.65–0.87), 0.04 (0.02–0.05), both P < 0.001. In females, the SI was not significantly associated with sarcopenia, OR and 95% CI were 0.99 (0.9 ~ 1.08), P = 0.775; for every 10 increase in SI, the SMI in females decreased by 0.03, β and 95% CI were -0.03 (-0.04 ~ -0.01), P = 0.001.

**Conclusion:**

In the Chinese community, the SI is negatively associated with sarcopenia in males and has moderate diagnostic test performance. It was not associated with sarcopenia in females, and using the SI to screen for sarcopenia in females may not be a reliable method.

**Supplementary Information:**

The online version contains supplementary material available at 10.1007/s41999-024-01111-w.

## Key summary points

**Aim:**

To analyse whether there are sex differences in the association between sarcopenia index and sarcopenia in a Chinese community-based population.

**Findings:**

In the community-based population, the sarcopenia index was significantly associated with sarcopenia in males and not significantly associated with sarcopenia in females.

**Message:**

Using the sarcopenia index to screen for sarcopenia may not be a reliable method in Chinese community-based female populations.

**Supplementary Information:**

The online version contains supplementary material available at 10.1007/s41999-024-01111-w.

## Introduction

Sarcopenia is a common chronic condition that is characterized by reduced skeletal muscle mass associated with a decrease in muscle strength or somatic function. According to the recommendations of the Asian Working Group for Sarcopenia 2019 (AWGS2019) [1], sarcopenia is defined as low muscle mass with low muscle strength or physical performance. The European Working Group on Sarcopenia in Older People 2 (EWGSOP2) recommends that sarcopenia is diagnosed when low muscle strength is combined with low muscle quantity or quality [2]. The prevalence of sarcopenia varies considerably between different populations, with studies reporting rates ranging from 7.8% to 69.5% in the middle-aged and older population [3]. Sarcopenia is associated with a reduction in quality of life and an increased risk of mortality. The early identification of sarcopenia and subsequent intervention are expected to improve the quality of life and survival rate of patients [4]. Currently, the diagnosis of sarcopenia is mainly based on dual-energy x-ray absorptiometry (DXA) and bioelectrical impedance analysis (BIA), which limits its value in community populations due to its high cost and complexity.

Creatinine is the product of muscle metabolism, the rate of creatinine production in the human body is relatively constant, with approximately 1 mg of creatinine per 20 g of muscle per day. However, in addition to the correlation with muscle mass, the level of blood creatinine is also influenced by numerous factors, including age, sex, and renal function status. Consequently, the use of blood creatinine alone to predict muscle mass is limited [5]. Cystatin C is a low-molecular-weight protein produced at a constant rate by all nucleated cells, is less affected by muscle mass, and is often used to assess glomerular filtration function [6]. Because creatinine and cystatin C have unique metabolic processes, the ratio of these two substances may more accurately reflect muscle mass and sarcopenia, and some scholars refer to this as the sarcopenia index (SI) [7].

The SI, as an easily available and relatively inexpensive biomarker, has been the subject of several studies analysing the value of its use in sarcopenia screening [3, 8, 9]. As creatinine differs significantly between sexes [5], while cystatin C is less affected by sex [6], this leads to differences in SI between sexes. Despite this, however, there is a lack of relevant studies addressing sex differences in the association between SI and sarcopenia. Moreover, most of the existing studies are based on specific populations, such as hospitalised patients [10], patients with malignant tumour [11] or chronic kidney disease [12], and intensive care unit (ICU) patients [13], making the findings potentially inapplicable to community populations. Therefore, in order to analyse the association between SI and sarcopenia in a community-based population and to determine whether sex differences exist, we conducted a cross-sectional study that included 7,118 participants from the China Health and Retirement Longitudinal Study (CHARLS).

## Methods

### Study population

The study population was drawn from the China Health and Retirement Longitudinal Study (CHARLS) baseline survey data from 2011. The survey randomly selected households with at least one member aged 45 years and older to be included in the study, and is generally representative of China's middle-aged and older community populations, as detailed information about CHARLS has been previously reported [14]. The baseline survey was collected between May 2011 and March 2012 by trained interviewers in participants' homes.

**Fig. 1:**
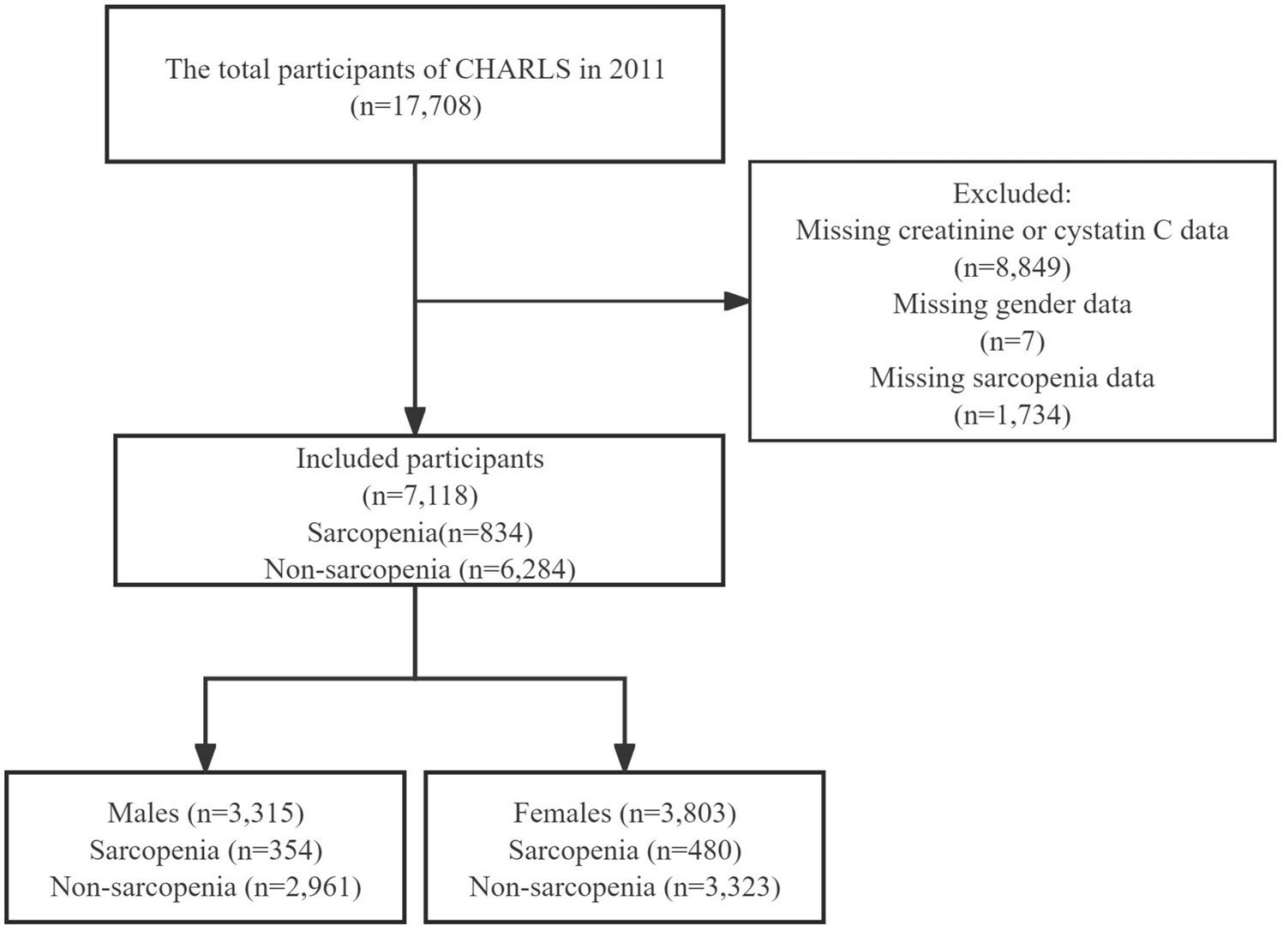
Flow chart of the screening of participants

In this cross-sectional study, participants were excluded if they lacked information on sarcopenia (n = 1,734), sex (n = 7), and creatinine or cystatin C (n = 8,849). A total of 7,118 participants were included in the analysis, and demographic characteristics between excluded and included participants are shown in Supplementary Table 1. The data were further analyzed separately by sex classification (Fig. 1).

The CHARLS study was approved by the Biomedical Ethics Committee of Peking University (approval number: IRB00001052-11015), and informed consent was obtained from each respondent prior to their participation in the survey [14]. This study was conducted in accordance with the relevant recommendations of the Strengthening the Reporting of Observational Studies in Epidemiology (STROBE) guideline for cross-sectional studies [15].

### Diagnose of sarcopenia

The diagnostic criteria for sarcopenia employed in this study were derived from the 2019 consensus of the AWGS2019 [1]: low skeletal muscle mass in conjunction with either low muscle strength or low physical performance.

As there were no data on DXA or BIA in CHARLS, we employed the muscle mass equations that have been demonstrated to exhibit satisfactory concordance with DXA in the Chinese population to calculate the appendicular skeletal muscle mass (ASM) of the participants [16]:

ASM = 0.193 × weight (kg) + 0.107 × height (cm) − 4.157 × sex − 0.037 × age (years) − 2.631.

In males, the value of sex is 1, in females, it is 2. The participants' height and weight were measured in the standing position using Seca™ 213 height meters (China Seca Hangzhou Co., Ltd.) and Omron™ HN-286 weight scales (Kerui Technology Yangzhou Co., Ltd.), measuring waist circumference using a soft ruler. Furthermore, the skeletal muscle mass index (SMI) was calculated as follows: SMI = ASM / height^2^. Referring to previous studies [17–19], the cut-off point for low skeletal muscle mass was set at the lowest 20% of sex-specific SMI in the study population: SMI < 6.96 kg/m^2^ in males and < 5.22 kg/m^2^ in females.

Muscle strength is assessed based on handgrip strength. Participants measured handgrip strength 2 times for each hand by using the Yuejian™ WL-1000 dynamometer (Nantong Yuejian Physical Testing Equipment Co., Ltd.), and low muscle strength was defined when the maximum handgrip strength was < 28 kg for males and < 18 kg for females.

Physical performance was assessed based on gait speed and 5 chair stand tests. Participants walked 1 round trip on a marked 2.5-m route for a total of 2 tests, using a stopwatch to record the walking time for the 2 tests and then further calculating gait speed. A 47-cm-high stool was used to perform the 5 chair stand tests, and a stopwatch was used to record the total time of the participants for the five repetitions of the sit-ups. Participants were considered to have low physical performance when their maximum gait speed was < 1 m/s or 5 chair stand tests ≥ 12 s.

### SI

Blood samples from all participants were collected by professionally trained blood collectors from the Chinese Center for Disease Control and Prevention, and the SI was calculated using the formula: SI = 100 × creatinine (mg/dL)/ cystatin C (mg/dL) [7].

### Covariates

The covariates included in our study included demographic characteristics, lifestyle, chronic diseases, and other blood test indicators. Specifically, demographic characteristics included: sex, age, education, marital status, and place of residence. Lifestyle included smoking status and alcohol consumption. Chronic disease data came from the questionnaire "Have you been diagnosed with a chronic disease by a doctor?" The results of the questionnaire include: hypertension, dyslipidemia, diabetes, cancer, chronic lung diseases, liver disease, heart disease, stroke, kidney disease, digestive disease, emotional problems, memory related disease, arthritis, and asthma. Other blood test data included blood urea nitrogen (BUN), triglycerides (TG), high-density lipoprotein cholesterol (HDL-c), low-density lipoprotein cholesterol (LDL-c), c-reactive protein (CRP), uric acid (UA), hemoglobin (HB), and estimated glomerular filtration rate (eGFR). High-risk cut-off points for blood test data are shown in Supplementary Table 2.

### Statistical analysis

All analyses were performed separately according to male and female sex. The SI after grouping by sex was processed by 4-quartile categorical transformation, where the males grouping was Q1 (≤ 72.19), Q2 (72.20 ~ 82.95), Q3 (82.96 ~ 94.83), and Q4 (≥ 94.84), and the females grouping was Q1 (≤ 62.78), Q2 (62.79 ~ 72.19), Q3 (72.20 ~ 82.04), and Q4 (≥ 82.05).

Continuous variables are expressed as mean ± standard deviation (SD) or median and interquartile range (IQR), respectively, and categorical variables are expressed as counts and percentages. Baseline characteristics of participants were compared using t-tests, ANOVA, chi-square tests, or other non-parametric tests. Across sex groups, we used logistic regression models to estimate odds ratios (ORs) and 95% confidence intervals (CIs) for sarcopenia. Linear regression modeling was used to assess the effects between SI with SMI, gait speed, 5 chair stand test, and handgrip strength. Natural spline models and inflection point analysis were used to test for linear or non-linear associations. Use of receiver operating characteristic (ROC) curves to assess the predictive power of the SI for sarcopenia in sex-specific participants.

Considering that eGFR and kidney disease significantly affect SI, we performed a sensitivity analysis. After excluding participants with eGFR < 30 ml/min/1.73 m^2^ or kidney disease, the association between SI and sarcopenia was analyzed again across sex to verify the stability of the results.

All the analyses were performed with the statistical software Stata/MP version 17.0 (StataCorp LP, College Station, TX, USA) and Free Statistics software versions 1.9.2. The level of statistical significance was set at P < 0.05 (two-sided).

## Results

### Baseline characteristics based on sex

We included 7,118 patients aged 60.6 ± 10.1 years; females were 53.4%. Of these, the overall prevalence of sarcopenia was 11.7%. Table 1 shows the baseline characteristics of the participants according to sex. Compared to females, males are older, more educated, more married, live more in rural areas, and are more likely to smoking and alcohol consumption. In terms of chronic diseases, males are less likely to suffer from hypertension, diabetes, dyslipidemia, heart disease, digestive disease, emotional disease, and arthritis, but more likely to suffer from chronic lung diseases, stroke, kidney disease, memory-related disease, and asthma. In terms of biomarkers, males possessed higher BUN, CRP, UA, HB, ASM, SMI, gait speed, handgrip strength, and lower TG, HDL-c, LDL-c, eGFR, and 5-time chair stand test. Notably, males had higher creatinine (0.9 ± 0.3 vs. 0.7 ± 0.2) and Cystatin C (1.1 ± 0.3 vs. 1.0 ± 0.3) than females. Since the sex difference in creatinine was greater than that of Cystatin C, the SI was higher in males than females (84.8 ± 20.0 vs. 74.2 ± 18.8, P < 0.001). The prevalence of sarcopenia was lower in males (10.7% vs. 12.6%, P = 0.011).

**Table 1:** Baseline characteristics of participants according to sex

	Total (n = 7118)	Female (n = 3803)	Male (n = 3315)	P-value
Age, year	60.6 ± 10.1	59.7 ± 10.4	61.7 ± 9.8	< 0.001
Education, n (%)				< 0.001
Primary school or below	5125 (72.0)	3020 (79.4)	2105 (63.5)	
Middle school	1319 (18.5)	523 (13.8)	796 (24)	
High school or above	674 ( 9.5)	260 (6.8)	414 (12.5)	
Marital, n (%)				< 0.001
Other	957 (13.4)	631 (16.6)	326 (9.8)	
Married	6161 (86.6)	3172 (83.4)	2989 (90.2)	
Residence, n (%)				0.003
Urban areas	1241 (17.4)	711 (18.7)	530 (16)	
Rural areas	5875 (82.6)	3091 (81.3)	2784 (84)	
Smoking status, n (%)				< 0.001
Never smoked	4334 (61.2)	3491 (92)	843 (25.6)	
Former smoker	639 ( 9.0)	81 (2.1)	558 (17)	
Current smoker	2110 (29.8)	222 (5.9)	1888 (57.4)	
Alcohol consumption, n (%)				< 0.001
Never or rarely	4352 (80.5)	3239 (91.5)	1113 (59.7)	
Less than once a month	555 (10.3)	206 (5.8)	349 (18.7)	
More than once a month	497 ( 9.2)	95 (2.7)	402 (21.6)	
Hypertension, n (%)	1841 (26.0)	1036 (27.4)	805 (24.4)	0.005
Dyslipidemia, n (%)	657 ( 9.4)	376 (10.1)	281 (8.7)	0.038
Diabetes, n (%)	425 ( 6.0)	257 (6.8)	168 (5.1)	0.003
Cancer, n (%)	60 ( 0.8)	38 (1)	22 (0.7)	0.123
Chronic lung diseases, n (%)	778 (11.0)	342 (9)	436 (13.2)	< 0.001
Liver disease, n (%)	270 ( 3.8)	129 (3.4)	141 (4.3)	0.057
Heart disease, n (%)	886 (12.5)	531 (14)	355 (10.8)	< 0.001
Stroke, n (%)	158 ( 2.2)	71 (1.9)	87 (2.6)	0.03
Kidney disease, n (%)	457 ( 6.5)	216 (5.7)	241 (7.3)	0.006
Digestive disease, n (%)	1691 (23.8)	959 (25.3)	732 (22.2)	0.002
Emotional disease, n (%)	102 ( 1.4)	67 (1.8)	35 (1.1)	0.012
Memory-related disease, n (%)	89 ( 1.3)	35 (0.9)	54 (1.6)	0.007
Arthritis, n (%)	2577 (36.3)	1515 (39.9)	1062 (32.2)	< 0.001
Asthma, n (%)	279 ( 3.9)	122 (3.2)	157 (4.8)	< 0.001
BUN, mg/dl	15.8 ± 4.6	15.1 ± 4.3	16.7 ± 4.8	< 0.001
TG, mg/dl	106.2 (75.2, 155.8)	114.2 (81.4, 162.8)	96.5 (69.9, 146.0)	< 0.001
HDL-c, mg/dl	50.9 ± 15.1	51.1 ± 14.1	50.7 ± 16.2	0.252
LDL-c, mg/dl	116.9 ± 35.4	120.5 ± 35.7	112.7 ± 34.6	< 0.001
CRP, mg/dl	1.1 (0.6, 2.2)	1.0 (0.5, 2.1)	1.1 (0.6, 2.3)	< 0.001
UA, mg/dl	4.5 ± 1.3	4.0 ± 1.1	5.0 ± 1.3	< 0.001
HB, g/dl	14.3 ± 2.2	13.6 ± 2.0	15.1 ± 2.2	< 0.001
eGFR, mL/(min × 1.73m^2^)	107.4 ± 29.4	110.6 ± 30.6	103.6 ± 27.5	< 0.001
Creatinine, mg/dl	0.8 ± 0.3	0.7 ± 0.2	0.9 ± 0.3	< 0.001
Cystatin C, mg/dl	1.0 ± 0.3	1.0 ± 0.3	1.1 ± 0.3	< 0.001
SI	79.2 ± 20.1	74.2 ± 18.8	84.8 ± 20.0	< 0.001
ASM, kg	17.0 ± 4.2	14.1 ± 2.7	20.4 ± 2.9	< 0.001
SMI, kg/m^2^	6.7 ± 1.1	6.0 ± 0.9	7.6 ± 0.8	< 0.001
Gait Speed, m/s	1.3 ± 0.5	1.3 ± 0.4	1.4 ± 0.5	< 0.001
5-Time Chair Stand Test, s	11.1 ± 4.5	11.5 ± 4.8	10.6 ± 4.1	< 0.001
Handgrip strength, kg	32.0 ± 10.3	26.5 ± 7.5	38.2 ± 9.6	< 0.001
Sarcopenia, n (%)	834 (11.7)	480 (12.6)	354 (10.7)	0.011

*BUN* blood urea nitrogen; *TG* total triglycerides; *HDL-c* high-density lipoprotein cholesterol; *LDL-c* low-density lipoprotein cholesterol; *CRP* high sensitivity C-reactive protein; *UA* uric acid; HB, hemoglobin; *eGFR* estimated glomerular filtration rate; *SI* Sarcopenia Index; *ASM* appendicular skeletal muscle mass; *SMI* skeletal muscle mass index

In males, compared with lower SI levels, participants with a higher SI were younger, had higher educational levels, more likely to live in cities, more married, non-smoking, more likely to have dyslipidemia and diabetes, less likely to have arthritis and asthma, and higher creatinine, TG, LDL-c, UA, HB, and lower eGFR, CRP, HDL-c, and Cystatin C, all P < 0.05. Males with higher SI had higher ASM, SMI, gait speed, handgrip strength, and lower time of 5-Time Chair Stand Test, all P < 0.001. The incidence of sarcopenia in males decreased with an increasing SI, P < 0.001 (Supplementary Table 3).

In females, trends in demographic characteristics, lifestyle, chronic diseases, blood test indicators, and sarcopenia were similar to those of males as SI levels increased (Supplementary Table 4).

### Association between SI and sarcopenia in different sexes.

In our cross-sectional study, the incidence of sarcopenia in the male Q1 to Q4 groups was 24.5%, 10.6%, 5.1%, and 2.5%; in the female Q1 to Q4 groups, it was 22.6%, 14.2%, 7.6%, and 6.2%. In univariate analyses (Supplementary Table 5), the results were similar for male and female participants. Specifically, the SI was significantly negatively correlated with the prevalence of sarcopenia and 5-time chair stand test; and it was significantly positively correlated with SMI, gait speed, and handgrip strength, both as continuous and categorical variables (all P < 0.001).

**Fig. 2:**
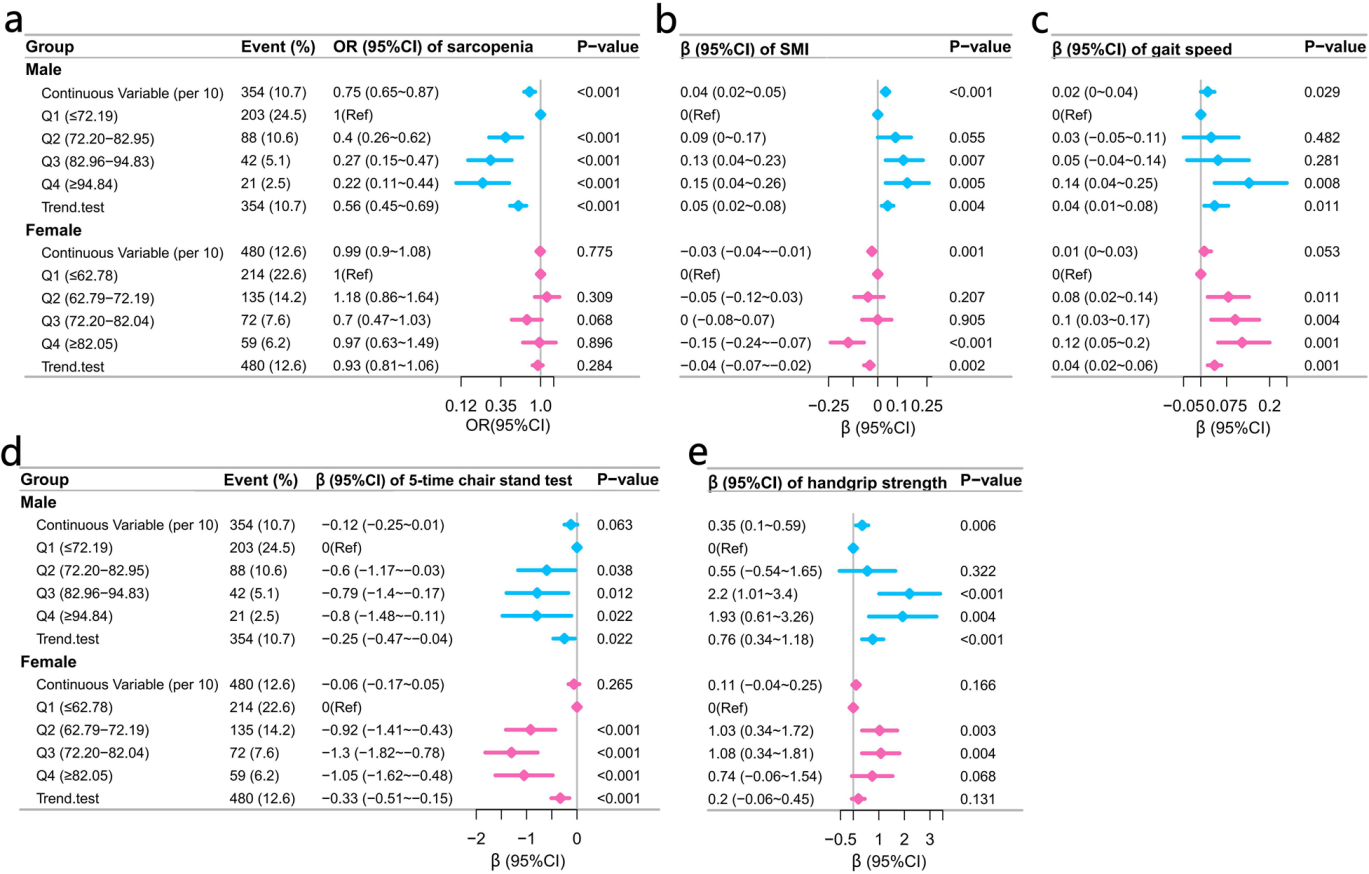
Multivariate analyses forest plot of the association of SI with sarcopenia (**a**), SMI (**b**), gait speed (**c**), 5-time chair stand test (**d**), and handgrip strength (**e**), with the SI as a continuous variable (per 10) and a categorical variable. The blue and pink colours represent males and females respectively, the squares represent OR or β, the lines represent 95% CI. Adjusted for: age, education, marital, residence, smoking, alcohol consumption, hypertension, dyslipidemia, diabetes, cancer, chronic lung diseases, liver disease, heart disease, stroke, kidney disease, digestive disease, emotional problems, memory related disease, arthritis, asthma, BUN, TG, HDL-c, LDL-c, CRP, UA, HB, eGFR. *OR* odds ratio; *CI* confidence interval; *SI* Sarcopenia Index; *SMI* skeletal muscle mass index; *BUN* blood urea nitrogen; *TG* total triglycerides; *HDL-c* high-density lipoprotein cholesterol; *LDL-c* low density lipoprotein cholesterol; *CRP* high sensitivity C-reactive protein; *UA* uric acid; *HB* hemoglobin; *eGFR* estimated glomerular filtration rate.

**Fig. 3:**
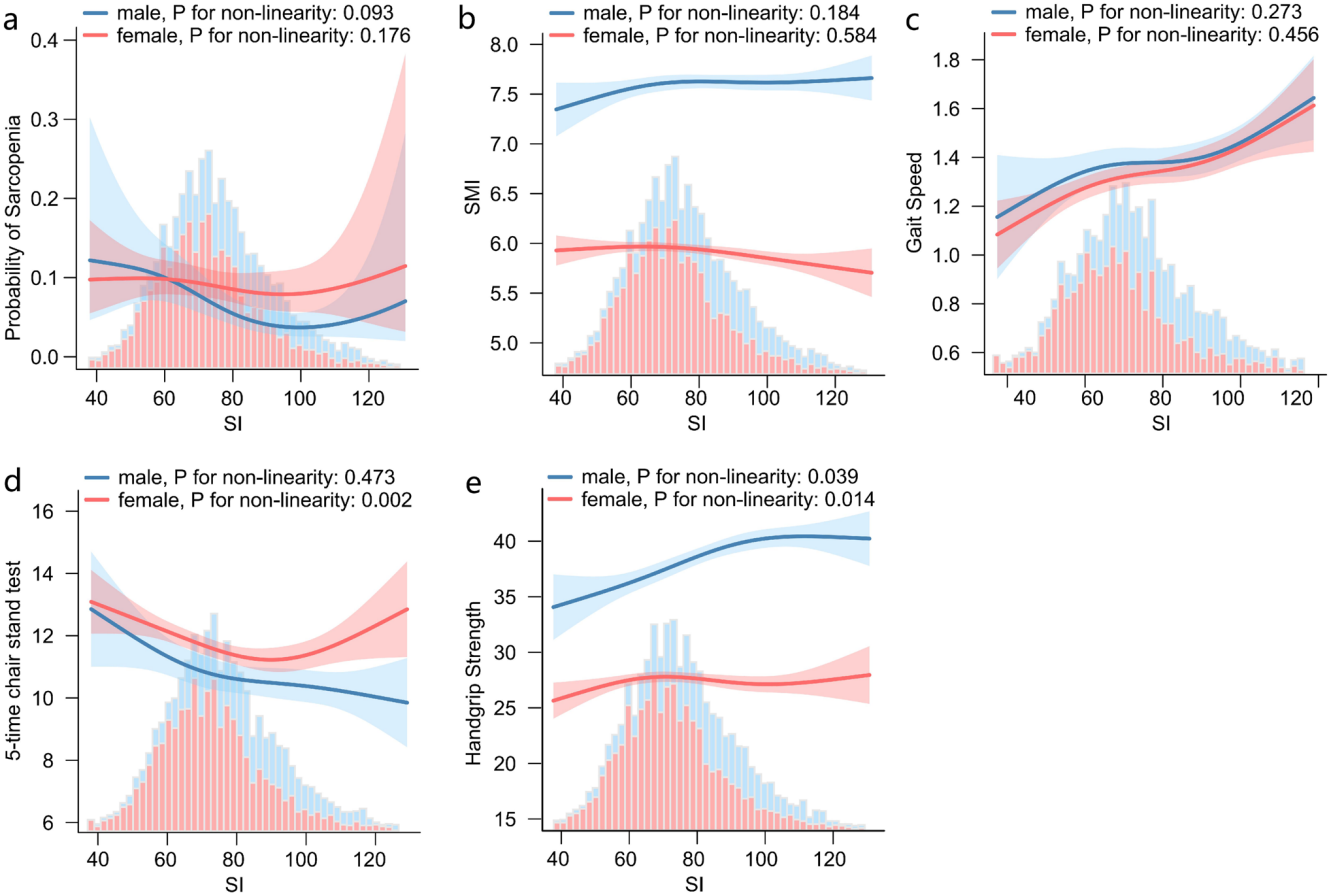
Natural spline model of the association of SI with sarcopenia (**a**), SMI (**b**), gait speed (**c**), 5-time chair stand test (**d**), and handgrip strength (**e**). The blue and pink colours represent males and females, respectively; the lines represent trends; and the coloured areas around them represent the 95% CI. Only 98% participants were included, with a knot of 4. Adjusted for: age, education, marital, residence, smoking, alcohol consumption, hypertension, dyslipidemia, diabetes, cancer, chronic lung diseases, liver disease, heart disease, stroke, kidney disease, digestive disease, emotional problems, memory related disease, arthritis, asthma, BUN, TG, HDL-c, LDL-c, CRP, UA, HB, eGFR. *SI* Sarcopenia Index; *SMI *skeletal muscle mass index; *CI* confidence interval; *BUN* blood urea nitrogen; *TG* total triglycerides; *HDL-c* high-density lipoprotein cholesterol; *LDL-c* low density lipoprotein cholesterol; *CRP* high sensitivity C-reactive protein; *UA* uric acid; *HB* hemoglobin; *eGFR* estimated glomerular filtration rate

The results of the multifactorial analyses are shown in Fig. 2 and Fig. 3. In males, SI was linearly negatively correlated with sarcopenia [OR and 95% CI: 0.75 (0.65 ~ 0.87), P < 0.001] and linearly positively correlated with SMI [β and 95% CI: 0.04 (0.02 ~ 0.05), P < 0.001] and gait speed [β and 95% CI: 0.02 (0 ~ 0.04), P = 0.029], all P for non-linearity > 0.05. The association between SI and handgrip strength in males was non-linear (P for non-linearity: 0.039), with an inflection point value around 95 [β and 95%CI for < 95: 0.87 (0.39 ~ 1.35), P < 0.001; for > 95: -0.21 (-1.08 ~ 0.66), P = 0.635]. The association between SI and 5-time chair stand test was not significant, β and 95%CI were -0.13 (-0.25 ~ 0), P = 0.063.

In the multivariate analysis of females, the SI was not significantly associated with sarcopenia [OR and 95% CI: 0.99 (0.9 ~ 1.08), P = 0.775], was linearly negatively associated with SMI [β and 95% CI: -0.03 (-0.04 ~ -0.01), P = 0.001], all P for non-linearity > 0.05. The association between SI and gait speed was not significant, β and 95% CI was 0.01 (0 ~ 0.03), P = 0.053. The association of SI with 5-time chair stand test and handgrip strength was non-linear, both P for non-linearity < 0.05. In the analysis with 5-time chair stand test, the inflection point value of the SI was around 89.27 [β and 95%CI for < 89.27: -0.39 (-0.6 ~ -0.18), P < 0.001; for > 89.27: 0.26 (-0.15 ~ 0.67), P = 0.21]. In the analyses with handgrip strength, the inflection point value was around 65.6 [β and 95% CI for < 65.6: 0.88 (0.12 ~ 1.64), P = 0.023; for > 65.6: -0.07 (-0.33 ~ 0.19), P = 0.603]. Supplementary Table 6 shows the results of the inflection point analysis for all non-linear relationships.

**Fig. 4:**
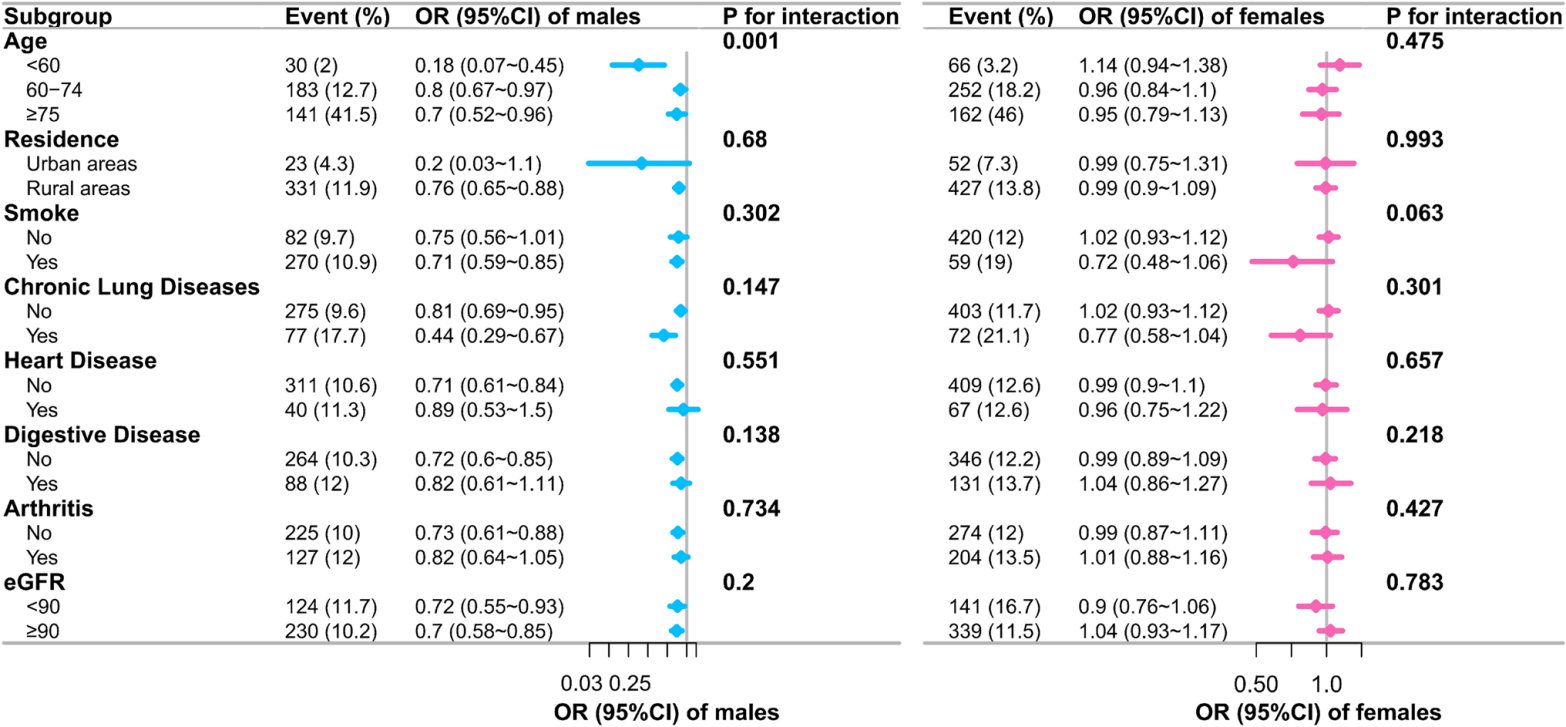
Subgroup forest plots of multifactorial logistic regression models for the association between SI and sarcopenia. The blue and pink colours represent males and females respectively, the squares represent OR and the lines represent 95% CI. Multivariate: adjusted for: age, education, marital, residence, smoking, alcohol consumption, hypertension, dyslipidemia, diabetes, cancer, chronic lung diseases, liver disease, heart disease, stroke, kidney disease, digestive disease, emotional problems, memory related disease, arthritis, asthma, BUN, TG, HDL-c, LDL-c, CRP, UA, HB, eGFR. *OR* odds ratio; *CI* confidence interval; *SI* Sarcopenia Index; *BUN* blood urea nitrogen; *TG* total triglycerides; *HDL-c* high-density lipoprotein cholesterol; *LDL-c* low density lipoprotein cholesterol; *CRP* high sensitivity C-reactive protein; *UA* uric acid; HB, hemoglobin; *eGFR* estimated glomerular filtration rate.

Figure 4 shows the results of a multivariate logistic regression model subgroup analysis of the association between SI and sarcopenia. The negative correlation between SI and sarcopenia became more pronounced in males aged < 60 (P for interaction = 0.001). Other than that, we did not find any other interactions.

**Fig. 5:**
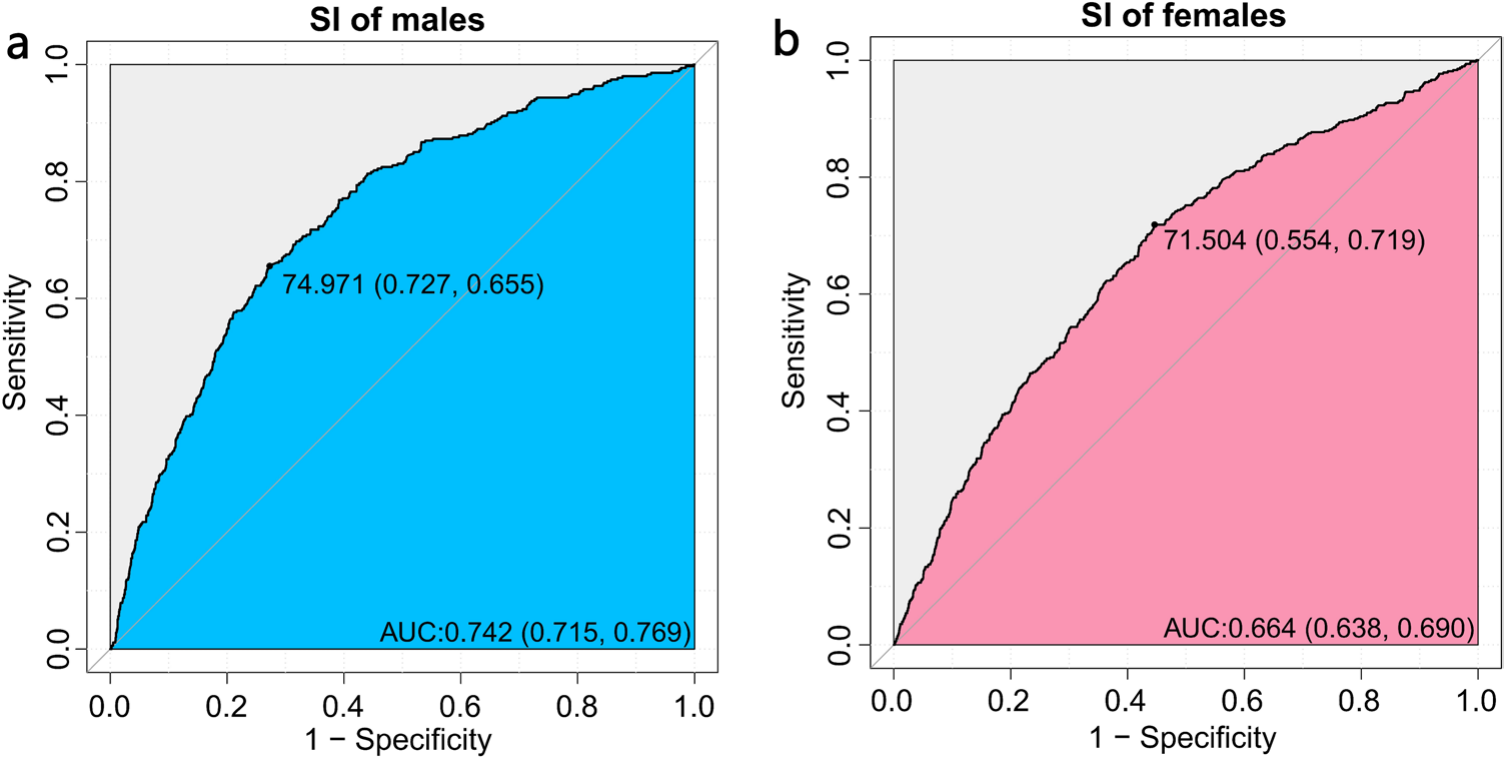
ROC curves for the sarcopenia index in males **a** an females, **b** *SI* sarcopenia index

Figure 5 shows the ROC curves for the association between SI and sarcopenia. The optimal threshold, specificity, and sensitivity of SI for males and females were 74.97 (0.727, 0.655) and 71.5 (0.554, 0.719), respectively, and the area under curve (AUC) was 0.742 and 0.664, respectively.

### Sex-based differences in SI across different sarcopenic traits.

In univariate analyses in males and females, low SMI, 5-time chair stand test ≥ 12 s, low walk speed, and low muscle strength were all associated with lower SI, all P < 0.001.

In the multivariate analysis of males, low SMI [β and 95% CI: -3.55 (-5.7 to -1.4), P = 0.001] and low muscle strength [β and 95% CI: -2.5 (-4.69 to -0.31), P = 0.026] were associated with a lower SI. There was no significant difference in SI across the 5-time chair stand test and walk speed. In the multifactorial analysis of females, SI was not significantly different across all sarcopenia traits, all P > 0.05. All of the above results are shown in Supplementary Table 7.

### Sensitivity analysis

The association between SI and sarcopenia was similar to previous results after excluding participants with eGFR < 30 ml/min/1.73 m^2^ ( male, n = 7; female, n = 2) or kidney disease (male, n = 265; female, n = 240) (Supplementary Table 8 and Supplementary Table 9).

## Discussion

This cross-sectional study used nationally representative data on adults in the CHARLS community, and the diagnosis of sarcopenia was based on the AWGS 2019 criteria. In our study, there were sex differences in the association between SI and sarcopenia. In multivariate analysis of males, SI was linearly positively correlated with SMI and gait speed, and linearly negatively correlated with sarcopenia; with a 25% decrease in the prevalence of sarcopenia for every 10 increase in SI. In females, SI was linearly negatively correlated with SMI, and had no significant association with sarcopenia.

The SI has been noted to reflect health status in a wide range of clinical practices [20], among several studies SI has been considered as a surrogate marker for sarcopenia [21–23]. In a meta-analysis [3], using the ratio of creatinine to cystatin C for the diagnosis of sarcopenia, the sensitivity, specificity, and AUC were 65%, 79%, and 0.78, respectively. However, sex was not included in the subgroup analysis of this study. A prospective cohort study including 312 hospitalized older adults showed that reduced SI had a high predictive value for both the diagnosis and poor prognosis of sarcopenia [10]. The SI, has also been reported to be a useful indicator for assessing muscle mass in intensive care unit patients [24]. In other specific populations, such as patients with chronic kidney disease [25], cardiovascular disease [26], and chronic obstructive pulmonary disease [27], sarcopenia is associated with a poor prognosis, and the SI can be used as surrogate marker for sarcopenia in the above mentioned patients. Unlike our study, in some of the above findings, there were no sex differences, while others were not analysed for the presence of sex differences.

There are also some other studies with results similar to ours. In a study of 327 gastric cancer patients, the SI was found to be a surrogate for sarcopenia in male gastric cancer patients, whereas no correlation was found in women [28]. Sex differences in results have been found in a number of studies on the association between SI and other diseases. In a cross-sectional study that included 166 females and 252 males, SI was negatively associated with osteoporosis in males, while there was no significant association in females [29]. In a study based on 1,926 CHARLS participants, a lower SI was associated with an increased prevalence of frailty in older males but not in older females [30]. In another CHARLS-based study, the SI was negatively associated with depression in males, with no significant association in females [31]. Although the aim of these studies was to analyse the association between SI and osteoporosis, frailty, and depression, to the best of our knowledge, all of these diseases are strongly associated with sarcopenia [32, 33].

In our ROC curves, the cut-off point, specificity, and sensitivity of SI were 74.97 (0.727, 0.655) and 71.5 (0.554, 0.719) for males and females with an AUC of 0.742 and 0.664, respectively. This suggests that in males SI has a moderate performance of diagnostic tests, while in females it is low. In a study by Fu X et al. for patients with advanced cancer [21], the cut-off point, specificity, and sensitivity of SI were 56 (0.876, 0.575) and 61.2 (0.333, 0.882), with AUCs of 0.764 and 0.603, respectively. There are some similarities with our results, and the SI seems to have better diagnostic test performance. However, in a study by Ding P et al. for hospitalized patients with gastrointestinal tumors [11], creatinine to cystatin C ratio possessed similar and higher performance in males and females, with cut-off points, specificity, and sensitivity of 0.65 (0.773, 0.777) and 0.65 (0.814, 0.799), and AUCs of 0.838 and 0.841, respectively. This suggests that the diagnostic test performance of the SI for sarcopenia may vary considerably depending on the study population.

In addition, our study further analysed the association between SI and SMI, gait speed, 5-time chair stand test, and handgrip strength, which together determine the diagnosis of sarcopenia. In particular, the association between SI and SMI showed significant sex differences. For every 10 increase in SI, SMI increased by 0.04 in males but decreased by 0.03 in females (Fig. 2b). Moreover, our study found that the SI was weakly correlated with muscle strength and physical performance in women than in men, and these factors ultimately led to a significant correlation between the SI and sarcopenia in men, but not in women. Similar to our study, several studies have also analysed the association between SI and muscle mass and muscle strength. A cross-sectional study that included 908 Japanese community-dwelling older adults showed that the ratio of creatinine to cystatin C was positively correlated with muscle mass [34]. The study by Lingling Tan et al., that included 1,098 Chinese community-dwelling males and 1,241 females, found that the SI was linearly and positively correlated with grip strength in both males and females [22]. In both studies, there were no sex differences in the results.

Age and sex may simultaneously influence the association between SI and sarcopenia. Compared to some previous studies [27, 34], our study population had a lower mean age (60.6 vs. 73.8 and 73.0). Therefore, in a subgroup analysis, we discussed whether the association between SI and sarcopenia differed among participants of different ages in different sexes. We found a stronger association between SI and sarcopenia in male participants aged < 60 years. There was no significant association between SI and sarcopenia in females of all ages. As a metabolite of muscle, the concentration of creatinine usually remains constant when muscle mass is stable. However, age, sex may cause fluctuations in creatinine even when muscle mass does not change [35, 36]. Cystatin C is secreted by all nucleated cells, and although it is usually maintained at a relatively stable level, sex can similarly affect cystatin C concentrations [6]. For these reasons, it is possible that the association between SI and sarcopenia may result in differences in populations with different sex and age. It has been shown that cystatin C mRNA is expressed in adipose tissue and that hypertrophied adipose tissue increases cystatin C production [37]. The mean age of the women included in our study was 59.7 ± 10.4, and most were postmenopausal. Changes in hormone levels after menopause altered the ratio of muscle mass to fat in female participants [38], which may affect the level of SI and its association with sarcopenia. However, further research is needed to analyse the reasons for this sex difference.

The strength of this study is the use of a large, nationally representative sample, which allows the findings to be generalised to community-based middle-aged and older populations in China. To the best of our knowledge, this is the first study to explore sex differences in the association between SI and sarcopenia based on the CHARLS. Moreover, our study further analysed the relationship between skeletal sarcopenia indices and muscle mass, muscle strength and physical performance across sexes. However, we should note some limitations. Firstly, although the formula for ASM has been validated in a Chinese population and has shown good agreement with DXA in various studies, muscle mass was estimated using the formula, not DXA or bioimpedance analysis. This is because there is no DXA or BIA data in CHARLS. Second, our study was limited to a Chinese community population and cannot be directly generalised to populations of other races or regions.

## Conclusions

In the Chinese community, the SI is negatively associated with sarcopenia in males and has moderate diagnostic test performance. However, it was not associated with sarcopenia in females, and using the SI to screen for sarcopenia in females may not be a reliable method.

## Supplementary Information

Below is the link to the electronic supplementary material.

Supplementary file1 (DOCX 80 KB)

## Data Availability

The data used in our study comes from China Health and Retirement Longitudinal Study (CHARLS), a publicly available database. This data can be found here: https://charls.pku.edu.cn/.
