# Supplementary material for: Sex differences in the association between sarcopenia index and sarcopenia: a cross-sectional study from a Chinese community-based population
Source: Eur Geriatr Med. 2024 Dec 2;16(1):55–65. doi: 10.1007/s41999-024-01111-w (PMC11850426; doi:10.1007/s41999-024-01111-w)
Supplement: Supplementary file 1 — Supplementary file1 (DOCX 80 KB) [file 41999_2024_1111_MOESM1_ESM.docx]

**Title:** Sex differences in the association between sarcopenia index and sarcopenia: a cross-sectional study from a Chinese community-based population.

**Author information：**Hong Yang^1a^, Yunda Huang^1a^, Guihua Jiang^1a^, Zhiping Duan^1^, Runfen Du^1^, Yinan Hao^1^, Wei Huang^1*^, Xiaoling Liu^2*^.

^1^Department of Geriatrics, The Third People's Hospital of Yunnan Province, Kunming, China;

^2^Radiotherapy Department, Yunnan Cancer Hospital, Kunming, China.

^a^These authors contributed equally to this work: Hong Yang, Yunda Huang, Guihua Jiang.

*Corresponding authors: Xiaoling Liu, Wei Huang.

Address correspondence to:

Xiaoling Liu, Radiotherapy Department, Yunnan Cancer Hospital, No. 519 Kunzhou Road, Kunming, Yunnan, 650118, China. Tel: +86 18387143003. Email: [liuxiaoling0725@126.com](mailto:liuxiaoling0725@126.com);

Wei Huang, Department of Geriatrics, The Third People's Hospital of Yunnan Province, 292 Beijing Road, Kunming, Yunnan, 650011, China. Tel: +86 13888111601. Email: Huangwei797@126.com.

ORCID：

Hong Yang: 0009-0003-0842-828X

Yunda Huang: 0009-0005-8892-8202

Xiaoling Liu: 0009-0000-3353-084X

**Supplementary Table 1.** Demographic characteristics between excluded and included participants.

|  | Total  (n = 17708) | Excluded  (n = 10590) | Included  (n = 7118) | P-value |
| --- | --- | --- | --- | --- |
| Sex, n (%) |  |  |  | 0.003 |
| Female | 8947 (52.1) | 5144 (51.1) | 3803 (53.4) |  |
| Male | 8231 (47.9) | 4916 (48.9) | 3315 (46.6) |  |
| Age, year | 59.2 ± 10.2 | 58.2 ± 10.0 | 60.6 ± 10.1 | < 0.001 |
| Education, n (%) |  |  |  | < 0.001 |
| Primary school or below | 11442 (66.7) | 6317 (62.9) | 5125 (72) |  |
| Middle school | 3541 (20.6) | 2222 (22.1) | 1319 (18.5) |  |
| High school or above | 2181 (12.7) | 1507 (15) | 674 (9.5) |  |
| Marital, n (%) |  |  |  | < 0.001 |
| Other | 2119 (12.3) | 1162 (11.6) | 957 (13.4) |  |
| Married | 15043 (87.7) | 8882 (88.4) | 6161 (86.6) |  |
| Residence, n (%) |  |  |  | < 0.001 |
| Urban areas | 4318 (24.5) | 3077 (29.2) | 1241 (17.4) |  |
| Rural areas | 13331 (75.5) | 7456 (70.8) | 5875 (82.6) |  |

**Supplementary Table 2.** High-risk cut-off points for blood test data.

|  | High-risk cut-off points |
| --- | --- |
| BUN | >20 mg/dl |
| TG | ≥240 mg/dl |
| HDL-cl | <40 mg/dl |
| LDL-c | >160 mg/dl |
| CRP | >3 mg/dl |
| HB | <13 g/dl for males; <12 g/dl for females |
| creatinine | >1.4 mg/dl |
| Cystatin C | >1.44 mg/dl |

Abbreviations: BUN, blood urea nitrogen; TG, total triglycerides; HDL-c, high-density lipoprotein cholesterol; LDL-c, low-density lipoprotein cholesterol; CRP, high sensitivity C-reactive protein; HB, hemoglobin.

**Supplementary table 3.** Baseline characteristics of males according to quartiles of SI.

|  | Total  (n = 3315) | Q1 (≤72.19)  (n = 828) | Q2 (72.20-82.95)  (n = 829) | Q3 (82.96-94.83)  (n = 827) | Q4 (≥94.84)  (n = 831) | P-value |
| --- | --- | --- | --- | --- | --- | --- |
| Age,year | 61.7 ± 9.8 | 66.0 ± 10.1 | 62.8 ± 9.5 | 60.2 ± 8.9 | 57.7 ± 8.6 | < 0.001 |
| Education, n (%) |  |  |  |  |  | < 0.001 |
| Primary school or below | 2105 (63.5) | 593 (71.6) | 542 (65.4) | 506 (61.2) | 464 (55.8) |  |
| Middle school | 796 (24.0) | 154 (18.6) | 198 (23.9) | 200 (24.2) | 244 (29.4) |  |
| High school or above | 414 (12.5) | 81 (9.8) | 89 (10.7) | 121 (14.6) | 123 (14.8) |  |
| Marital, n (%) |  |  |  |  |  | < 0.001 |
| Other | 326 ( 9.8) | 111 (13.4) | 97 (11.7) | 68 (8.2) | 50 (6) |  |
| Married | 2989 (90.2) | 717 (86.6) | 732 (88.3) | 759 (91.8) | 781 (94) |  |
| Residence, n (%) |  |  |  |  |  | < 0.001 |
| Urban areas | 530 (16.0) | 77 (9.3) | 110 (13.3) | 157 (19) | 186 (22.4) |  |
| Rural areas | 2784 (84.0) | 751 (90.7) | 719 (86.7) | 669 (81) | 645 (77.6) |  |
| Smoking status, n (%) |  |  |  |  |  | 0.002 |
| Never smoked | 843 (25.6) | 184 (22.5) | 201 (24.4) | 214 (26.1) | 244 (29.4) |  |
| Former smoker | 558 (17.0) | 140 (17.1) | 126 (15.3) | 132 (16.1) | 160 (19.3) |  |
| Current smoker | 1888 (57.4) | 493 (60.3) | 497 (60.3) | 473 (57.8) | 425 (51.3) |  |
| Alcohol consumption, n (%) |  |  |  |  |  | 0.059 |
| Never or rarely | 1113 (59.7) | 316 (63.6) | 296 (60.3) | 258 (59) | 243 (55.4) |  |
| Less than once a month | 349 (18.7) | 90 (18.1) | 100 (20.4) | 73 (16.7) | 86 (19.6) |  |
| More than once a month | 402 (21.6) | 91 (18.3) | 95 (19.3) | 106 (24.3) | 110 (25.1) |  |
| Hypertension, n (%) | 805 (24.4) | 183 (22.2) | 213 (25.8) | 200 (24.4) | 209 (25.3) | 0.341 |
| Dyslipidemia, n (%) | 281 ( 8.7) | 54 (6.7) | 60 (7.4) | 76 (9.4) | 91 (11.2) | 0.005 |
| Diabetes, n (%) | 168 ( 5.1) | 28 (3.4) | 37 (4.5) | 47 (5.8) | 56 (6.8) | 0.013 |
| Cancer, n (%) | 22 ( 0.7) | 7 (0.9) | 6 (0.7) | 4 (0.5) | 5 (0.6) | 0.822 |
| Chronic lung diseases, n (%) | 436 (13.2) | 130 (15.8) | 111 (13.4) | 100 (12.2) | 95 (11.5) | 0.057 |
| Liver disease, n (%) | 141 ( 4.3) | 38 (4.6) | 39 (4.7) | 34 (4.2) | 30 (3.7) | 0.691 |
| Heart disease, n (%) | 355 (10.8) | 93 (11.3) | 87 (10.5) | 87 (10.6) | 88 (10.6) | 0.949 |
| Stroke, n (%) | 87 ( 2.6) | 15 (1.8) | 22 (2.7) | 27 (3.3) | 23 (2.8) | 0.313 |
| Kidney disease, n (%) | 241 ( 7.3) | 48 (5.9) | 60 (7.3) | 74 (9) | 59 (7.2) | 0.103 |
| Digestive disease, n (%) | 732 (22.2) | 174 (21.1) | 176 (21.3) | 205 (25) | 177 (21.4) | 0.175 |
| Emotional disease, n (%) | 35 ( 1.1) | 12 (1.5) | 7 (0.8) | 10 (1.2) | 6 (0.7) | 0.441 |
| Memory-related disease, n (%) | 54 ( 1.6) | 20 (2.4) | 13 (1.6) | 13 (1.6) | 8 (1) | 0.136 |
| Arthritis, n (%) | 1062 (32.2) | 297 (36) | 248 (30) | 262 (31.8) | 255 (30.8) | 0.04 |
| Asthma, n (%) | 157 ( 4.8) | 51 (6.2) | 47 (5.7) | 36 (4.4) | 23 (2.8) | 0.005 |
| BUN, mg/dl | 16.7 ± 4.8 | 16.8 ± 4.8 | 16.4 ± 4.6 | 16.6 ± 4.6 | 16.8 ± 5.2 | 0.305 |
| TG, mg/dl | 96.5 (69.9, 146.0) | 84.5 (64.6, 114.2) | 95.6 (69.0, 133.6) | 97.3 (69.0, 141.6) | 122.1 (80.5, 197.4) | < 0.001 |
| HDL-c, mg/dl | 50.7 ± 16.2 | 53.0 ± 16.8 | 51.0 ± 15.9 | 50.9 ± 15.6 | 47.8 ± 16.0 | < 0.001 |
| LDL-c, mg/dl | 112.7 ± 34.6 | 106.0 ± 33.0 | 111.8 ± 33.3 | 115.5 ± 34.5 | 117.6 ± 36.6 | < 0.001 |
| CRP, mg/dl | 1.1 (0.6, 2.3) | 1.3 (0.6, 3.4) | 1.2 (0.6, 2.5) | 0.9 (0.5, 1.9) | 1.0 (0.6, 2.0) | < 0.001 |
| UA, mg/dl | 5.0 ± 1.3 | 4.8 ± 1.2 | 4.9 ± 1.3 | 5.0 ± 1.2 | 5.3 ± 1.3 | < 0.001 |
| HB, g/dl | 15.1 ± 2.2 | 14.7 ± 2.5 | 15.0 ± 2.0 | 15.3 ± 2.1 | 15.4 ± 2.0 | < 0.001 |
| eGFR, mL/(min×1.73m^2^) | 103.6 ± 27.5 | 120.6 ± 34.0 | 104.4 ± 23.8 | 98.4 ± 20.6 | 91.2 ± 20.3 | < 0.001 |
| creatinine, mg/dl | 0.9 ± 0.3 | 0.8 ± 0.2 | 0.9 ± 0.2 | 0.9 ± 0.2 | 1.0 ± 0.5 | < 0.001 |
| Cystatin C, mg/dl | 1.1 ± 0.3 | 1.2 ± 0.3 | 1.1 ± 0.2 | 1.0 ± 0.2 | 0.9 ± 0.4 | < 0.001 |
| ASM, kg | 20.4 ± 2.9 | 19.2 ± 2.9 | 20.0 ± 2.7 | 20.8 ± 2.6 | 21.4 ± 2.8 | < 0.001 |
| SMI, kg/m^2^ | 7.6 ± 0.8 | 7.3 ± 0.8 | 7.5 ± 0.7 | 7.7 ± 0.7 | 7.9 ± 0.7 | < 0.001 |
| Gait Speed, m/s | 1.4 ± 0.5 | 1.3 ± 0.5 | 1.3 ± 0.4 | 1.4 ± 0.5 | 1.5 ± 0.5 | < 0.001 |
| 5-Time Chair Stand Test, s | 10.6 ± 4.1 | 11.6 ± 4.9 | 10.8 ± 3.8 | 10.2 ± 3.7 | 9.7 ± 3.4 | < 0.001 |
| Handgrip strength, kg | 38.2 ± 9.6 | 34.4 ± 9.6 | 37.1 ± 9.1 | 39.7 ± 9.2 | 41.7 ± 8.6 | < 0.001 |
| Sarcopenia, n (%) | 354 (10.7) | 203 (24.5) | 88 (10.6) | 42 (5.1) | 21 (2.5) | < 0.001 |

Abbreviations: SI, Sarcopenia Index; BUN, blood urea nitrogen; TG, total triglycerides; HDL-c, high-density lipoprotein cholesterol; LDL-c, low-density lipoprotein cholesterol; CRP, high sensitivity C-reactive protein; UA, uric acid; HB, hemoglobin; eGFR, estimated glomerular filtration rate; ASM, appendicular skeletal muscle mass; SMI, skeletal muscle mass index.

**Supplementary Table 4.** Baseline characteristics of females according to quartiles of SI

|  | Total  (n = 3803) | Q1 (≤62.78)  (n = 949) | Q2 (62.79-72.19)  (n = 951) | Q3 (72.20-82.04)  (n = 950) | Q4 (≥82.05)  (n = 953) | P-value |
| --- | --- | --- | --- | --- | --- | --- |
| Age,year | 59.7 ± 10.4 | 64.6 ± 10.9 | 60.9 ± 9.8 | 57.4 ± 9.1 | 56.0 ± 9.4 | < 0.001 |
| Education, n (%) |  |  |  |  |  | < 0.001 |
| Primary school or below | 3020 (79.4) | 832 (87.7) | 807 (84.9) | 722 (76) | 659 (69.2) |  |
| Middle school | 523 (13.8) | 81 (8.5) | 94 (9.9) | 154 (16.2) | 194 (20.4) |  |
| High school or above | 260 ( 6.8) | 36 (3.8) | 50 (5.3) | 74 (7.8) | 100 (10.5) |  |
| Marital, n (%) |  |  |  |  |  | < 0.001 |
| Other | 631 (16.6) | 261 (27.5) | 152 (16) | 107 (11.3) | 111 (11.6) |  |
| Married | 3172 (83.4) | 688 (72.5) | 799 (84) | 843 (88.7) | 842 (88.4) |  |
| Residence, n (%) |  |  |  |  |  | < 0.001 |
| Urban areas | 711 (18.7) | 152 (16) | 159 (16.7) | 173 (18.2) | 227 (23.8) |  |
| Rural areas | 3091 (81.3) | 796 (84) | 792 (83.3) | 777 (81.8) | 726 (76.2) |  |
| Smoking status, n (%) |  |  |  |  |  | 0.037 |
| Never smoked | 3491 (92.0) | 851 (89.8) | 867 (91.6) | 881 (92.8) | 892 (93.9) |  |
| Former smoker | 81 ( 2.1) | 24 (2.5) | 24 (2.5) | 16 (1.7) | 17 (1.8) |  |
| Current smoker | 222 ( 5.9) | 73 (7.7) | 56 (5.9) | 52 (5.5) | 41 (4.3) |  |
| Alcohol consumption, n (%) |  |  |  |  |  | 0.908 |
| Never or rarely | 3239 (91.5) | 796 (91.8) | 811 (91) | 810 (91) | 822 (92.2) |  |
| Less than once a month | 206 ( 5.8) | 47 (5.4) | 53 (5.9) | 58 (6.5) | 48 (5.4) |  |
| More than once a month | 95 ( 2.7) | 24 (2.8) | 27 (3) | 22 (2.5) | 22 (2.5) |  |
| Hypertension, n (%) | 1036 (27.4) | 264 (28.1) | 278 (29.4) | 231 (24.4) | 263 (27.7) | 0.091 |
| Dyslipidemia, n (%) | 376 (10.1) | 86 (9.4) | 85 (9.1) | 92 (9.9) | 113 (12.1) | 0.136 |
| Diabetes, n (%) | 257 ( 6.8) | 46 (4.9) | 61 (6.5) | 72 (7.7) | 78 (8.2) | 0.023 |
| Cancer, n (%) | 38 ( 1.0) | 9 (1) | 10 (1.1) | 8 (0.8) | 11 (1.2) | 0.914 |
| Chronic lung diseases, n (%) | 342 ( 9.0) | 102 (10.8) | 83 (8.8) | 83 (8.8) | 74 (7.8) | 0.136 |
| Liver disease, n (%) | 129 ( 3.4) | 27 (2.9) | 36 (3.8) | 28 (3) | 38 (4) | 0.412 |
| Heart disease, n (%) | 531 (14.0) | 136 (14.5) | 151 (15.9) | 122 (12.9) | 122 (12.9) | 0.17 |
| Stroke, n (%) | 71 ( 1.9) | 29 (3.1) | 17 (1.8) | 18 (1.9) | 7 (0.7) | 0.003 |
| Kidney disease, n (%) | 216 ( 5.7) | 60 (6.4) | 54 (5.7) | 58 (6.1) | 44 (4.6) | 0.379 |
| Digestive disease, n (%) | 959 (25.3) | 230 (24.3) | 261 (27.5) | 243 (25.6) | 225 (23.7) | 0.233 |
| Emotional disease, n (%) | 67 ( 1.8) | 13 (1.4) | 27 (2.9) | 14 (1.5) | 13 (1.4) | 0.036 |
| Memory-related disease, n (%) | 35 ( 0.9) | 9 (0.9) | 13 (1.4) | 7 (0.7) | 6 (0.6) | 0.345 |
| Arthritis, n (%) | 1515 (39.9) | 423 (44.7) | 373 (39.3) | 359 (37.8) | 360 (38) | 0.006 |
| Asthma, n (%) | 122 ( 3.2) | 48 (5.1) | 32 (3.4) | 22 (2.3) | 20 (2.1) | < 0.001 |
| BUN, mg/dl | 15.1 ± 4.3 | 15.1 ± 4.5 | 15.3 ± 4.2 | 15.0 ± 4.2 | 14.9 ± 4.5 | 0.273 |
| TG, mg/dl | 114.2 (81.4, 162.8) | 105.3 (78.8, 138.9) | 112.4 (80.5, 153.1) | 114.2 (77.0, 164.6) | 137.2 (91.2, 209.7) | < 0.001 |
| HDL-c, mg/dl | 51.1 ± 14.1 | 52.7 ± 14.7 | 52.0 ± 13.5 | 51.1 ± 14.0 | 48.5 ± 13.8 | < 0.001 |
| LDL-c, mg/dl | 120.5 ± 35.7 | 117.0 ± 34.1 | 123.1 ± 36.6 | 120.6 ± 33.8 | 121.4 ± 37.9 | 0.002 |
| CRP, mg/dl | 1.0 (0.5, 2.1) | 1.2 (0.6, 2.4) | 1.1 (0.6, 2.3) | 0.9 (0.5, 1.9) | 0.9 (0.5, 1.9) | < 0.001 |
| UA, mg/dl | 4.0 ± 1.1 | 4.0 ± 1.1 | 4.0 ± 1.1 | 4.0 ± 1.1 | 4.2 ± 1.1 | < 0.001 |
| HB, g/dl | 13.6 ± 2.0 | 13.5 ± 2.1 | 13.7 ± 2.0 | 13.7 ± 2.1 | 13.8 ± 1.9 | 0.004 |
| eGFR, mL/(min×1.73m^2^) | 110.6 ± 30.6 | 128.0 ± 41.4 | 110.1 ± 24.7 | 106.5 ± 22.1 | 97.8 ± 21.5 | < 0.001 |
| creatinine, mg/dl | 0.7 ± 0.2 | 0.6 ± 0.2 | 0.7 ± 0.1 | 0.7 ± 0.1 | 0.8 ± 0.2 | < 0.001 |
| Cystatin C, mg/dl | 1.0 ± 0.3 | 1.1 ± 0.3 | 1.0 ± 0.2 | 0.9 ± 0.2 | 0.8 ± 0.2 | < 0.001 |
| ASM, kg | 14.1 ± 2.7 | 13.3 ± 2.8 | 13.9 ± 2.6 | 14.4 ± 2.5 | 14.6 ± 2.5 | < 0.001 |
| SMI, kg/m^2^ | 6.0 ± 0.9 | 5.8 ± 1.0 | 5.9 ± 0.8 | 6.1 ± 0.8 | 6.2 ± 0.8 | < 0.001 |
| Gait Speed, m/s | 1.3 ± 0.4 | 1.2 ± 0.4 | 1.3 ± 0.4 | 1.3 ± 0.4 | 1.3 ± 0.5 | < 0.001 |
| 5-Time Chair Stand Test, s | 11.5 ± 4.8 | 13.0 ± 6.1 | 11.7 ± 5.0 | 10.8 ± 3.4 | 10.8 ± 4.2 | < 0.001 |
| Handgrip strength, kg | 26.5 ± 7.5 | 23.9 ± 7.3 | 26.4 ± 7.2 | 27.7 ± 7.4 | 28.1 ± 7.3 | < 0.001 |
| Sarcopenia, n (%) | 480 (12.6) | 214 (22.6) | 135 (14.2) | 72 (7.6) | 59 (6.2) | < 0.001 |

Abbreviations: SI, Sarcopenia Index; BUN, blood urea nitrogen; TG, total triglycerides; HDL-c, high-density lipoprotein cholesterol; LDL-c, low-density lipoprotein cholesterol; CRP, high sensitivity C-reactive protein; UA, uric acid; HB, hemoglobin; eGFR, estimated glomerular filtration rate; ASM, appendicular skeletal muscle mass; SMI, skeletal muscle mass index.

**Supplementary Table 5.** ORs, βs, and 95% CIs for sarcopenia (OR), SMI (β_1_), gait speed (β_2_), 5-time chair stand test (β_3_), and handgrip strength (β_4_), with the SI as a continuous variable (per 10) and a categorical variable in univariate analysis.

|  | Cases (%) | Univariate | | | | |
| --- | --- | --- | --- | --- | --- | --- |
|  |  | OR (95 % CI) | β_1_ (95 % CI) | β_2_ (95 % CI) | β_3_ (95 % CI) | β_4_ (95 % CI) |
| **Male** |  |  |  |  |  |  |
| Continuous Variable  (per 10) | 354 (10.7) | 0.59 (0.55~0.64)*** | 0.11 (0.09~0.12)*** | 0.03 (0.02~0.04)*** | -0.33 (-0.4~-0.26)*** | 1.27 (1.11~1.43)*** |
| Q1  (≤72.19) | 203 (24.5) | 1(Ref) | 0(Ref) | 0(Ref) | 0(Ref) | 0(Ref) |
| Q2  (72.20-82.95) | 88 (10.6) | 0.37 (0.28~0.48)*** | 0.21 (0.14~0.28)*** | 0.02 (-0.04~0.08) | -0.86 (-1.25~-0.46)*** | 2.72 (1.84~3.61)*** |
| Q3  (82.96-94.83) | 42 (5.1) | 0.16 (0.12~0.23)*** | 0.39 (0.32~0.46)*** | 0.09 (0.03~0.15)** | -1.4 (-1.8~-1)*** | 5.34 (4.45~6.22)*** |
| Q4  (≥94.84) | 21 (2.5) | 0.08 (0.05~0.13)*** | 0.56 (0.49~0.63)*** | 0.19 (0.12~0.25)*** | -1.89 (-2.29~-1.49)*** | 7.33 (6.44~8.22)*** |
| Trend.test | 354 (10.7) | 0.41 (0.37~0.47)*** | 0.19 (0.16~0.21)*** | 0.06 (0.04~0.08)*** | -0.62 (-0.75~-0.49)*** | 2.46 (2.18~2.74)*** |
| **Female** |  |  |  |  |  |  |
| Continuous Variable  (per 10) | 480 (12.6) | 0.7 (0.66~0.75)*** | 0.07 (0.05~0.08)*** | 0.02 (0.01~0.03)*** | -0.31 (-0.4~-0.23)*** | 0.71 (0.59~0.84)*** |
| Q1  (≤62.78) | 214 (22.6) | 1(Ref) | 0(Ref) | 0(Ref) | 0(Ref) | 0(Ref) |
| Q2  (62.79-72.19) | 135 (14.2) | 0.57 (0.45~0.72)*** | 0.16 (0.09~0.24)*** | 0.11 (0.06~0.16)*** | -1.33 (-1.78~-0.88)*** | 2.46 (1.8~3.12)*** |
| Q3  (72.20-82.04) | 72 (7.6) | 0.28 (0.21~0.37)*** | 0.34 (0.26~0.41)*** | 0.15 (0.09~0.21)*** | -2.16 (-2.61~-1.72)*** | 3.78 (3.11~4.44)*** |
| Q4  (≥82.05) | 59 (6.2) | 0.23 (0.17~0.31)*** | 0.37 (0.29~0.45)*** | 0.17 (0.11~0.23)*** | -2.21 (-2.65~-1.76)*** | 4.19 (3.53~4.85)*** |
| Trend.test | 480 (12.6) | 0.58 (0.53~0.64)*** | 0.13 (0.1~0.15)*** | 0.06 (0.04~0.08)*** | -0.74 (-0.88~-0.6)*** | 1.39 (1.18~1.6)*** |

Abbreviations: SI, Sarcopenia Index; OR, odds ratio; CI, confidence interval; SMI, skeletal muscle mass index.

* P < 0.05

** P < 0.01

*** P < 0.001

**Supplementary Table 6.** Results of inflection point analysis after adjusting for all covariates.

|  | handgrip strength for males | |  | 5-time chair stand test for females | |  | handgrip strength for females | |
| --- | --- | --- | --- | --- | --- | --- | --- | --- |
|  | inflection point. β (95%CI) | P-value |  | inflection point. β (95%CI) | P-value |  | inflection point. β (95%CI) | P-value |
| SI | 95 |  |  | 89.27(88.6~89.9) |  |  | 65.6(65.1~66.2) |  |
| slope1 (per 10) | 0.87(0.39~1.35) | <0.001 |  | -0.39(-0.6~-0.18) | <0.001 |  | 0.88(0.12~1.64) | 0.023 |
| slope2 (per 10) | -0.21(-1.08~0.66) | 0.635 |  | 0.26(-0.15~0.67) | 0.21 |  | -0.07(-0.33~0.19) | 0.603 |
| Likelihood Ratio test | - | 0.032 |  | - | 0.016 |  | - | 0.035 |
| Non-linear Test*1 | - | 0.043 |  | - | 0.002 |  | - | 0.013 |
| Non-linear Test*2 | - | 0.023 |  | - | 0.002 |  | - | 0.013 |

Adjusted for all covariates: age, education, marital, residence, smoking, alcohol consumption, hypertension, dyslipidemia, diabetes, cancer, chronic lung diseases, liver disease, heart disease, stroke, kidney disease, digestive disease, emotional problems, memory related disease, arthritis, asthma, BUN, TG, HDL-c, LDL-c, CRP, UA, HB, eGFR.

Abbreviations: SI, Sarcopenia Index; CI, confidence interval; BUN, blood urea nitrogen; TG, total triglycerides; HDL-c, high-density lipoprotein cholesterol; LDL-c, low density lipoprotein cholesterol; CRP, high sensitivity C-reactive protein; UA, uric acid; HB, hemoglobin; eGFR, estimated glomerular filtration rate.

**Supplementary Table 7.** Linear regression modeling of sex-based differences in SI across sarcopenia traits.

|  | n | Univariate |  |  | Multivariate |  |
| --- | --- | --- | --- | --- | --- | --- |
|  |  | β (95 % CI) | P-value |  | β (95 % CI) | P-value |
| **Male** |  |  |  |  |  |  |
| Normal SMI | 2746 | 0(Ref) |  |  | 0(Ref) |  |
| Low SMI | 569 | -12.91 (-14.67~-11.16) | <0.001 |  | -3.55 (-5.7~-1.4) | 0.001 |
| 5-Time Chair Stand Test <12s | 2284 | 0(Ref) |  |  | 0(Ref) |  |
| 5-Time Chair Stand Test ≥12s | 866 | -5.74 (-7.28~-4.2) | <0.001 |  | -1.37 (-3.03~0.29) | 0.106 |
| Normal walk speed | 1401 | 0(Ref) |  |  | 0(Ref) |  |
| Low walk speed | 357 | -4.98 (-7.02~-2.93) | <0.001 |  | -1.86 (-4.22~0.5) | 0.123 |
| Normal muscle strength | 2843 | 0(Ref) |  |  | 0(Ref) |  |
| Low muscle strength | 428 | -11.28 (-13.28~-9.28) | <0.001 |  | -2.5 (-4.69~-0.31) | 0.026 |
| **Female** |  |  |  |  |  |  |
| Normal SMI | 3168 | 0(Ref) |  |  | 0(Ref) |  |
| Low SMI | 635 | -8.25 (-9.83~-6.67) | <0.001 |  | 1.33 (-0.33~3) | 0.116 |
| 5-Time Chair Stand Test <12s | 2255 | 0(Ref) |  |  | 0(Ref) |  |
| 5-Time Chair Stand Test ≥12s | 1308 | -4.65 (-5.9~-3.4) | <0.001 |  | -0.99 (-2.14~0.16) | 0.093 |
| Normal walk speed | 1227 | 0(Ref) |  |  | 0(Ref) |  |
| Low walk speed | 498 | -2.06 (-3.96~-0.16) | 0.034 |  | -0.82 (-2.61~0.98) | 0.372 |
| Normal muscle strength | 3327 | 0(Ref) |  |  | 0(Ref) |  |
| Low muscle strength | 416 | -7.22 (-9.12~-5.32) | <0.001 |  | -0.8 (-2.57~0.96) | 0.374 |

Multivariate: adjusted for all covariates: age, education, marital, residence, smoking, alcohol consumption, hypertension, dyslipidemia, diabetes, cancer, chronic lung diseases, liver disease, heart disease, stroke, kidney disease, digestive disease, emotional problems, memory related disease, arthritis, asthma, BUN, TG, HDL-c, LDL-c, CRP, UA, HB, eGFR.

Abbreviations: SI, Sarcopenia Index; CI, confidence interval; SMI, skeletal muscle mass index; BUN, blood urea nitrogen; TG, total triglycerides; HDL-c, high-density lipoprotein cholesterol; LDL-c, low density lipoprotein cholesterol; CRP, high sensitivity C-reactive protein; UA, uric acid; HB, hemoglobin; eGFR, estimated glomerular filtration rate.

**Supplementary Table 8.** ORs, βs, and 95% CIs for sarcopenia (OR), SMI (β_1_), gait speed (β_2_), 5-time chair stand test (β_3_), and handgrip strength (β_4_), with the SI as a continuous variable (per 10) and a categorical variable in univariate analysis. After excluding participants with eGFR <30 ml/min/1.73 m^2^ or kidney disease.

|  | Cases (%) | Univariate | | | | |
| --- | --- | --- | --- | --- | --- | --- |
|  |  | OR (95 % CI) | β_1_ (95 % CI) | β_2_ (95 % CI) | β_3_ (95 % CI) | β_4_ (95 % CI) |
| **Male** |  |  |  |  |  |  |
| Continuous Variable  (per 10) | 323 (10.6) | 0.58 (0.54~0.63)*** | 0.11 (0.1~0.12)*** | 0.03 (0.02~0.05)*** | -0.34 (-0.41~-0.27)*** | 1.3 (1.14~1.47)*** |
| Q1  (≤72.19) | 188 (24.4) | 1(Ref) | 0(Ref) | 0(Ref) | 0(Ref) | 0(Ref) |
| Q2  (72.20-82.95) | 78 (10.2) | 0.35 (0.27~0.47)*** | 0.23 (0.16~0.31)*** | 0.02 (-0.04~0.08) | -0.83 (-1.24~-0.41)*** | 2.85 (1.93~3.77)*** |
| Q3  (82.96-94.83) | 40 (5.4) | 0.18 (0.12~0.25)*** | 0.4 (0.32~0.47)*** | 0.1 (0.03~0.16)** | -1.41 (-1.82~-0.99)*** | 5.39 (4.46~6.32)*** |
| Q4  (≥94.84) | 17 (2.2) | 0.07 (0.04~0.12)*** | 0.59 (0.51~0.66)*** | 0.19 (0.12~0.26)*** | -1.91 (-2.32~-1.49)*** | 7.45 (6.52~8.37)*** |
| Trend.test | 323 (10.6) | 0.41 (0.36~0.47)*** | 0.19 (0.17~0.21)*** | 0.06 (0.04~0.08)*** | -0.63 (-0.76~-0.5)*** | 2.49 (2.2~2.78)*** |
| **Female** |  |  |  |  |  |  |
| Continuous Variable  (per 10) | 450 (12.6) | 0.69 (0.64~0.74)*** | 0.07 (0.05~0.08)*** | 0.02 (0.01~0.04)*** | -0.33 (-0.42~-0.24)*** | 0.72 (0.59~0.85)*** |
| Q1  (≤62.78) | 202 (22.9) | 1(Ref) | 0(Ref) | 0(Ref) | 0(Ref) | 0(Ref) |
| Q2  (62.79-72.19) | 123 (13.9) | 0.54 (0.42~0.69)*** | 0.17 (0.09~0.25)*** | 0.11 (0.06~0.17)*** | -1.45 (-1.92~-0.99)*** | 2.6 (1.91~3.29)*** |
| Q3  (72.20-82.04) | 69 (7.8) | 0.28 (0.21~0.38)*** | 0.35 (0.28~0.43)*** | 0.14 (0.08~0.2)*** | -2.24 (-2.7~-1.77)*** | 3.77 (3.08~4.46)*** |
| Q4  (≥82.05) | 56 (6.2) | 0.22 (0.16~0.3)*** | 0.38 (0.3~0.46)*** | 0.17 (0.1~0.23)*** | -2.29 (-2.75~-1.83)*** | 4.21 (3.53~4.9)*** |
| Trend.test | 450 (12.6) | 0.58 (0.53~0.64)*** | 0.13 (0.11~0.16)*** | 0.06 (0.04~0.08)*** | -0.76 (-0.9~-0.61)*** | 1.38 (1.16~1.59)*** |

Abbreviations: SI, Sarcopenia Index; OR, odds ratio; CI, confidence interval; SMI, skeletal muscle mass index; eGFR, estimated glomerular filtration rate.

* P < 0.05

** P < 0.01

*** P < 0.001

**Supplementary Table 9.** ORs, βs, and 95% CIs for sarcopenia (OR), SMI (β_1_), gait speed (β_2_), 5-time chair stand test (β_3_), and handgrip strength (β_4_), with the SI as a continuous variable (per 10) and a categorical variable in multivariate analysis. After excluding participants with eGFR <30 ml/min/1.73 m^2^ or kidney disease.

|  | Cases (%) | Multivariate | | | | |
| --- | --- | --- | --- | --- | --- | --- |
|  |  | OR (95 % CI) | β_1_ (95 % CI) | β_2_ (95 % CI) | β_3_ (95 % CI) | β_4_ (95 % CI) |
| **Male** |  |  |  |  |  |  |
| Continuous Variable  (per 10) | 323 (10.6) | 0.73 (0.62~0.85)*** | 0.04 (0.02~0.06)*** | 0.02 (0~0.05) | -0.14 (-0.28~0) | 0.35 (0.08~0.61)* |
| Q1  (≤72.19) | 188 (24.4) | 1(Ref) | 0(Ref) | 0(Ref) | 0(Ref) | 0(Ref) |
| Q2  (72.20-82.95) | 78 (10.2) | 0.39 (0.25~0.63)*** | 0.12 (0.03~0.21)** | 0.02 (-0.07~0.11) | -0.55 (-1.14~0.04) | 0.8 (-0.34~1.94) |
| Q3  (82.96-94.83) | 40 (5.4) | 0.31 (0.18~0.55)*** | 0.16 (0.06~0.26)** | 0.04 (-0.05~0.14) | -0.79 (-1.44~-0.14)* | 2.02 (0.77~3.28)** |
| Q4  (≥94.84) | 17 (2.2) | 0.18 (0.08~0.41)*** | 0.19 (0.09~0.3)*** | 0.13 (0.01~0.24)* | -0.88 (-1.59~-0.16)* | 2.15 (0.77~3.53)** |
| Trend.test | 323 (10.6) | 0.56 (0.44~0.7)*** | 0.06 (0.03~0.1)** | 0.04 (0~0.07)* | -0.28 (-0.51~-0.06)* | 0.77 (0.33~1.21)** |
| **Female** |  |  |  |  |  |  |
| Continuous Variable  (per 10) | 480 (12.6) | 0.96 (0.87~1.06) | -0.02 (-0.04~-0.01)** | 0.02 (0~0.03)* | -0.05 (-0.16~0.06) | 0.1 (-0.05~0.26) |
| Q1  (≤62.78) | 214 (22.6) | 1(Ref) | 0(Ref) | 0(Ref) | 0(Ref) | 0(Ref) |
| Q2  (62.79-72.19) | 135 (14.2) | 1.13 (0.81~1.59) | -0.04 (-0.11~0.04) | 0.08 (0.02~0.15)** | -0.93 (-1.44~-0.42)*** | 1.17 (0.45~1.88)** |
| Q3  (72.20-82.04) | 72 (7.6) | 0.68 (0.45~1.02) | 0.02 (-0.06~0.09) | 0.1 (0.03~0.17)** | -1.25 (-1.79~-0.72)*** | 1.13 (0.37~1.89)** |
| Q4  (≥82.05) | 59 (6.2) | 0.95 (0.61~1.49) | -0.14 (-0.23~-0.06)** | 0.12 (0.05~0.2)** | -0.97 (-1.56~-0.39)** | 0.76 (-0.07~1.58) |
| Trend.test | 480 (12.6) | 0.92 (0.8~1.06) | -0.04 (-0.07~-0.01)** | 0.04 (0.02~0.06)** | -0.29 (-0.48~-0.11)** | 0.18 (-0.08~0.45) |

Multivariate: adjusted for all covariates: age, education, marital, residence, smoking, alcohol consumption, hypertension, dyslipidemia, diabetes, cancer, chronic lung diseases, liver disease, heart disease, stroke, kidney disease, digestive disease, emotional problems, memory related disease, arthritis, asthma, BUN, TG, HDL-c, LDL-c, CRP, UA, HB, eGFR.

Abbreviations: SI, Sarcopenia Index; CI, confidence interval; BUN, blood urea nitrogen; TG, total triglycerides; HDL-c, high-density lipoprotein cholesterol; LDL-c, low density lipoprotein cholesterol; CRP, high sensitivity C-reactive protein; UA, uric acid; HB, hemoglobin; eGFR, estimated glomerular filtration rate.

* P < 0.05

** P < 0.01

*** P < 0.001
